# Wnt Receptor Frizzled-4 as a Marker for Isolation of Enteric Neural Progenitors in Human Children

**DOI:** 10.3390/cells8080792

**Published:** 2019-07-30

**Authors:** Peter H. Neckel, Melanie Scharr, Karin Seid, Katharina Nothelfer, Jörg Fuchs, Florian Obermayr, Bernhard Hirt, Stephan M. Huber, Lothar Just

**Affiliations:** 1 Institute of Clinical Anatomy and Cell Analysis, University of Tübingen, 72074 Tübingen, Germany; 2 Department of Pediatric Surgery, University Children’s Hospital, 72076 Tübingen, Germany; 3 Department of Pediatric Surgery, University Children’s Hospital, 35043 Marburg, Germany; 4 Department of Radiation Oncology, University of Tübingen, 72076 Tübingen, Germany

**Keywords:** enteric nervous system, frizzled, Wnt, progenitor cells, stem cell marker, human stem cells, FACS

## Abstract

Identification and isolation of neural progenitor cells from the human enteric nervous system (ENS) is currently hampered by the lack of reliable, specific markers. Here, we define the Wnt-receptor frizzled-4 as a marker for the isolation of enteric neural progenitor cells derived from paediatric gut samples. We show that the Wnt-receptor frizzled-4 is expressed in the human colon and in *Tunica muscularis*-derived enterospheres. To obtain a purified culture, we carried out fluorescence-activated cell sorting (FACS) using PE-conjugated frizzled-4 antibodies. Frizzled-4^positive^ cells gave rise to neurosphere-like bodies and ultimately differentiated into neurons as revealed by BrdU-proliferation assays and immunocytochemistry, whereas in frizzled-4^negative^ cultures we did not detect any neuronal and glial cells. By using a patch-clamp approach, we also demonstrated the expression of functional sodium and potassium channels in frizzled-4^positive^ cell cultures after differentiation in vitro.

## 1. Introduction

The enteric nervous system (ENS) is a complex network of neurons and glial cells organized into two dominating ganglionated plexus within the alimentary tract. Its complex histoarchitecture enables the ENS to influence and control vital functions of the gastrointestinal tract, often autonomously, and independent of higher central processing [1]. A missing or malfunctioning ENS causes severe pathologies, including achalasia [2], idiopathic gastroparesis [3], or Hirschsprung’s disease [4], all of which have high impact on quality of life and are prone to fatal complications.

In search of new treatments for such enteric neuropathies, the characterization of neural stem or progenitor cells from the postnatal ENS, as a potential source for future cell replacement therapies, made a lot of progress during the last two decades [5,6]. After initial reports of proliferative neural cells in the postnatal intestine of rats by Kruger et al. [7], ENS progenitors were isolated from mice [8,9] and human gut samples [10], even from elderly patients [11]. Although these cells exhibit a decreasing proliferative capacity over the course of aging [5], they can be expanded and differentiated in vitro to give rise to glial cells and different neuronal subtypes. To aid identification or isolation of ENS progenitors, several partly species-specific markers, e.g., p75^NTR^ [7], integrins [7], RET [9], nestin [11], sox2 [12], sox10 [9], HNK-1 [13], were tested and combined with fluorescence-activated cell sorting (FACS) to generate purified culture systems. Most commonly used is the neurotrophin-receptor p75^NTR^. All of these markers are especially expressed in the neural crest or in the course of development, which makes them particularly useful for studying ENS organogenesis [14]. Often, however, their expression in the postnatal and adult intestine can be found in mature neurons and glial cells [5], fibrocytes/-blasts [15], or immune cells [16]. The identification of novel markers to enrich neural progenitor cells is therefore a worthwhile goal for ongoing studies.

Wnt signalling is a well-conserved inter-cell communication system, playing major roles in the morphogenesis and development of several organ systems [17]. It is also established that Wnt pathways are crucially involved in the regulation of proliferation and differentiation of stem cell compartments (e.g., in the intestinal epithelium or the subventricular zone) and that its dysregulation can cause cancer [18,19]. Within the last few years, evidence accumulated indicating that Wnt signalling does not only play a role during the development of the ENS but can also enhance proliferation of neural ENS progenitors in vitro [20,21,22,23,24]. Moreover, our previous immunohistological study shows the co-expression of the Wnt-receptor frizzled-4 (Fzd4) with the putative stem cell markers nestin and the neurotrophin-receptor P75^NTR^ in the human ENS. Flow-cytometric analysis verified that frizzled-4^positive^ cells represent a subpopulation of the P75^NTRpositive^ cell pool [25].

In this short communication, we evaluated if frizzled-4 is useful as a marker for the FACS-based isolation of enteric neural progenitor cells from human resectates. Therefore, we compared the expression of frizzled-4 and p75^NTR^ in tissue sections of the human colon and in *Tunica muscularis*-derived enterospheres. We also used FACS-purification to quantify the frizzled-4^positive^ cell pool and characterized frizzled-4^positive^ cells with BrdU incorporation assays and immunohistochemistry. Additionally, we analysed the electrophysiologic properties of frizzled-4^positive^ cultures using patch-clamp technique.

## 2. Materials and Methods

### 2.1. Human Specimen

Human gut samples were obtained from 13 male and female patients aged one to 40 months, who were surgically treated due to imperforate anus, yolk sac tumour, meconium ileus, meconium plug syndrome or Hirschsprung’s disease. The sparse samples were collected over the course of two years and are summarized in Supplementary Table S1. All samples were collected after approval of the local ethical committee and with the consent of the patients’ parents (project number: 035/2015BO2).

### 2.2. Nomenclature: Enterosphere vs. Neurosphere

In this report we refer to unsorted *Tunica muscularis*-derived spheroids as enterospheres. These enterospheres are composed of all cell types that can be found within the *Tunica muscularis* including non-neural cells.

For neurospheres, we used FACS-based purification using anti-frizzled-4 antibodies prior to cell culture to exclude non-neural cells.

### 2.3. Isolation, FACS, and Culture of Enteric Neurospheres

The intestinal tissue was cut open along the longitudinal axis and rinsed twice with human preparation medium (HBSS without Ca^2+^/Mg^2+^ (Sigma-Aldrich, St. Louis, MO, USA) supplemented with penicillin (100 U/mL; PAA, Cambridge, UK), streptomycin (100 µg/mL; Sigma-Aldrich), ciprofloxacin (5 µm/mL; Fresenius Kabi, Bad Homburg, Germany), and metronidazole (50 µg/mL; B. Braun, Melsungen, Germany). The *Tunica adventitia* and scar tissue were removed, and the *Tunica muscularis* was peeled off the *Tela submucosa*. *Tunica muscularis* preparations were then stored in human preparation medium at 4 °C overnight. The day after, the *Tunica muscularis* was chopped multiple times (800 µm each) using a Mcllwain tissue chopper (Mickle Laboratory Engineering CO, Guildford, UK). The pieces were then transferred to collagenase type XI (750 U/mL; Sigma-Aldrich) and dispase II (250 µg/mL; Roche Diagnostics, Mannheim, Germany) containing 0.05% (*w*/*v*) DNase I (Sigma-Aldrich) and incubated for no longer than 60 min at 37 °C. Tissue was carefully triturated every 15 min with a fire polished 25 mL serological pipette. After tissue dissociation, fetal calf serum (FCS; Biochrom, Berlin, Germany) was added to a concentration of 10% (*v*/*v*). The cells were pelleted at 200 g and erythrocyte lysis was performed using RBC Lysis buffer (eBioscience, Frankfurt a.M., Germany). After a second centrifucation at 200 *g*, the pellet was resuspended in HBSS without Ca^2+^/Mg^2+^ (HBSS; Sigma-Aldrich) and filtered using 500, 200, and 70 µm cell strainers. Cells were pelleted again at 200 g and resuspended in human proliferation medium (Dulbecco’s modified Eagle’s medium with Ham’s F12 medium (DMEM/F12; 1:1; Life technologies, Darmstadt, Germany) containing N2 supplement (1:100; Life technologies, Darmstadt, Germany), penicillin (100 U/mL)/streptomycin (100 µg/mL; Sigma-Aldrich), ciprofloxacin (5 µg/mL; Fresenius Kabi), L-glutamine (2 mM; Sigma-Aldrich), epidermal growth factor (EGF; 20 ng/mL; Sigma-Aldrich), and fibroblast growth factor (bFGF; 20 ng/mL; Sigma-Aldrich)). Cell suspension was then filtered using a 30 µm cell strainer and pretreated with 20 µl of 10% (*v*/*v*) polyglobin (Talecris Biotherapeutics, Frankfurt am Main, Germany), 0.5% BSA in PBS per 10^6^ cells and incubated on ice for 20 min. Then, 25 µl cell culture medium per 10^6^ cells supplemented with Y-27632 (Merck KGaA, Darmstadt, Germany, 10 mM) and EGF/FGF (20 ng/mL) was added to the suspension. For the staining of frizzled-4 we used a mouse anti-frizzled-4 PE-conjugated antibody (Biolegend, San Diego, USA, 1:50). Cells were then washed with cell culture medium and centrifuged for 6 min and 200 *g*. The resulting pellet was re-suspended in cell culture medium supplemented with Y-27632 (10 mM), EGF/FGF (20 ng/mL), and B27 supplement (1:50).

FACS was performed with a BD FACS Aria flow cytometer (BD Biosciences, Franklin Lakes, NJ, USA) using a 100 μm nozzle. Forward-sideward-scatter dot plots were used to exclude debris and cell aggregates. PE was excited by a 488 nm laser. Emission filter was 576/26 nm. Data was analysed using FlowJo software (FlowJo LLC, Ashland, OR, USA).

After FACS, purified cells were seeded into multi-well plates (Greiner Bio-One GmbH, Frickenhausen, Germany) in a concentration of 4.0 × 10^4^ counts/cm^2^. The medium was supplemented with B27 (1:50; Life technologies), Y-27632 (10 mM) and Wnt3a (100 ng/mL). EGF and FGF were added daily and culture medium was exchanged every 5 days. Under proliferation conditions, purified Frizzled-4^positive^ cells formed three dimensional free-floating spheres. The cells were kept in proliferative cell culture for two weeks in total.

Differentiation of spheres was induced by transferring them into differentiation culture DMEM/F12 medium containing N2 supplement (1:100), penicillin (100 U/mL)/streptomycin (100 µg/mL), L-glutamine (2 mM), Ciprofloxacin Kabi (5 µg/mL), and ascorbic acid-2-phosphate (200 μM Sigma-Aldrich). Enterospheres were not dissociated before reseeding them. To facilitate attachment of neurospheres, culturing dishes were coated with collagen type I (5 µg/cm^2^, BD Biosciences, Heidelberg, Germany) and 2% (*v*/*v*) FCS was added to the differentiation cell culturing medium. Enterospheres quickly attached and flattened during the differentiation phase. All cultivation steps were conducted in a humidified incubator at 37 °C and 5% CO_2_. Cells were kept under differentiation conditions for one week in total. For patch-clamping experiments, the cells were differentiated for four weeks.

### 2.4. Immunohistochemistry

Cell cultures and 12–14 µm thick cryosections were fixated with 4% (*w*/*v*) phosphate buffered *p*-formaldehyde (PFA; Sigma-Aldrich) for 20 min and rinsed three times with phosphate buffered saline (PBS). To prevent unspecific binding of antibodies, samples were blocked for 30 min with PBS containing 4% (*v*/*v*) goat serum (Biochrom, Berlin, Germany), 0.1% (*v*/*v*) bovine serum albumin (BSA; Roth, Karlsruhe, Germany), and 0.1% (*v*/*v*) Triton^®^ X-100 (Roth) followed by incubation of primary antibody diluted in PBS with 0.1% (*v*/*v*) BSA and 0.1% (*v*/*v*) Triton^®^ X-100 overnight at 4 °C. For BrdU detection, cells were pretreated with 2 N HCl (Roth) at 37 °C for 30 min in a humidity chamber.

The antibodies used in this study are summarized in Supplementary Table S2. Nuclear 2.4. staining was carried out with 4′,6-diamidino-2-phenylindole (DAPI) solution (200 ng/mL, Roth). A Zeiss Axio Imager.Z1 fluorescence microscope (Zeiss, Jena, Germany) was used for microscopic evaluation. Images were acquired using Zeiss ZEN software (Zeiss).

### 2.5. Patch-Clamp Recording

For whole-cell recording (Figure 4), expanded frizzled-4^postive^ cells were seeded in a collagen type I coated petri dish and held for at least 4 weeks under differentiation conditions. Whole-cell currents were evoked by pulse protocols depicted in Figure 4A,E. Voltages were corrected for the estimated liquid junction potentials. Cells were superfused at 37 °C with NaCl solution (in mM: 125 NaCl, 32 *N*-*2*-hydroxyethylpiperazine-*N*-*2*-ethanesulfonic acid (HEPES), 5 KCl, 5 d-glucose, 1 MgCl_2_, 1 CaCl_2_, 0 or 3 mM tetraethylammonium (TEA) titrated with NaOH to pH 7.4). The pipette solution contained (in mM) 140 K-d-gluconate, 5 HEPES, 5 MgCl_2_, 1 K_2_-EGTA, 1 K_2_-ATP, titrated with KOH to pH 7.4.

## 3. Results and Discussion

### 3.1. Frizzled-4 Expression in Tunica Muscularis-Derived Enterospheres

Enteric neural progenitor cells react to a Wnt3a stimulus by an increased proliferation and with a higher yield of new-born neurons in vitro [24]. In a previous study, we showed that this effect is caused by the activation of the canonical Wnt pathway (e.g., by Wnt3a), which is downstream of frizzled and lipoprotein receptor-related proteins (LRP) receptors [24]. Extensive ligand-receptor-interaction studies also indicate that frizzled-4 and Wnt3a bind with high affinity to initiate the canonical Wnt pathway [26]. Additionally, we found that frizzled-4 is expressed by some mature neurons and glial cells but also by a subpopulation of cells expressing the potential stem cell marker P75^NTR^ [25] and also exhibits a considerable co-expression with nestin in human enteric ganglia [25]. Thus, we hypothesize that frizzled-4 is expressed by putative neural progenitors of the human ENS and might be a more precise marker than the commonly used P75^NTR^ stainings.

**Figure 1:**
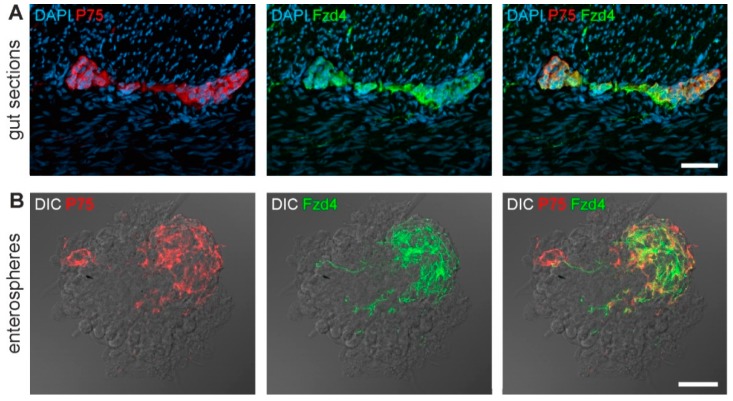
Expression of Fzd4 and P75^NTR^ in vivo and in *Tunica muscularis*-derived enterospheres. The micrographs show immunostainings for Fzd4 (green) and P75^NTR^ (red) in cryosections from *Tunica muscularis* (**A**) and *Tunica muscularis*-derived enterospheres (**B**). Fzd4 is expressed in ganglion cell bodies and neurites within the muscle sheets. There is a pronounced co-expression with the putative stem cell marker P75^NTR^. In enterospheres (spontaneously formed, non-purified), the same co-localization can be observed. Moreover, these stainings also illustrate the mixture of different cell types that can be found in unpurified enterospheres, thereby resembling a *Tunica muscularis*-like microenvironment. Immunohistochemistry in enterospheres is overlayed with differential interference contrast (DIC) images for better orientation. Scale bars: top panels 50 µm, bottom panels 40 µm.

The present study confirmed our previous findings [25] showing the expression of frizzled-4 and p75^NTR^ in myenteric ganglia in cryosections of the human colon (Figure 1A). We found considerable expression of both markers within the ganglia (also with a large overlap), but no immune-reactivity in surrounding muscle layers.

Moreover, we isolated cells from the *Tunica muscularis* of human intestinal resectates to generate three-dimensional free-floating enterospheres in an in vitro culture for 14 days. After collecting the spheroids, we performed cryosections and subsequently immunostainings for p75^NTR^ and frizzled-4. As Figure 1B shows, a subpopulation of cells within these enterospheres expressed at least one of the two markers, again with a large overlap (Figure 1B). This finding is therefore a first evidence for frizzled-4^positive^ cells within enterospheres. Such human enterospheres have repeatedly shown to give rise to newborn neurons [10,11,24]. Further, these expression patterns also shed light on the complex mixture of cell types present inside spontaneously generating spheroids, partly resembling the studies by Binder and colleagues [27].

### 3.2. FACS Isolation of Frizzled-4^positive^ Cells

**Figure 2:**
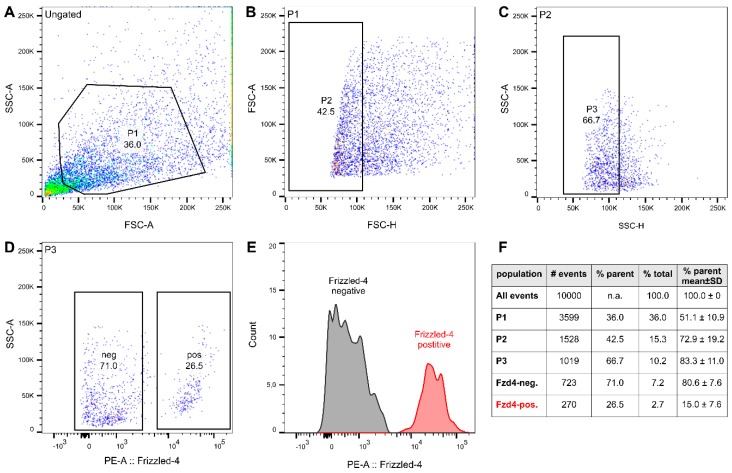
Fluorescence-activated cell sorting (FACS) sorting of Fzd4 marked cells derived from human *Tunica muscularis*. Shown is the scatter blot with sequential gates (Ungated, P1–P3; **A**–**C**) as well as gates marked for Fzd4^−^ and Fzd4^+^ cell pools (**D**,**E**) of one representative FACS experiment. The table (**F**) summarizes the number of counted events, the percentage of the parent population and of the total count of this representative experiment. In addition, the mean percentage of the parent population of five independent experiments is shown in the last column (mean ± STD, *n* = 5). In all FACS experiments, the Fzd4^+^ cell pool was a clearly distinct population.

In order to obtain purified spheroids containing exclusively frizzled-4^positive^ cells, we stained cell suspensions from human *Tunica muscularis* with a PE-conjugated anti-frizzled-4 antibody and subsequent used fluorescence-activated cell sorting (FACS). Similar to our previous results by Nothelfer et al. [25], we found that frizzled-4^positive^ cells make up a numerically stable (15.0 ± 7.6% of counts in parent population in five independent patients, Figure 2) and very distinct cell pool, clearly distinguishable from the rest of the *Tunica muscularis* cell population (Figure 2). As previously shown, these cells are furthermore a subpopulation of the P75^NTRpositive^ cell population [25], which is commonly used for the isolation of enteric neural progenitors [27,28]. We also carried out ancestry analyses of frizzled-4^negative^ (Supplementary Figure S1A) and frizzled-4^positive^ (Supplementary Figure S1B) to assess whether these populations differ from the main population or exhibits a specific scatter profile. We found, however, that there was no reliable correlation in between different patients. Together, the flow cytometry analysis supports our findings that frizzled-4 expression defines a clearly distinguishable cell population and together with P75^NTR^ shares an overlapping expression pattern, both in sections from the gut wall as well as in *Tunica muscularis*-derived enterospheres.

### 3.3. Frizzled-4^positive^ Cells Give Rise to Neurospheres and New-Born Neurons

**Figure 3:**
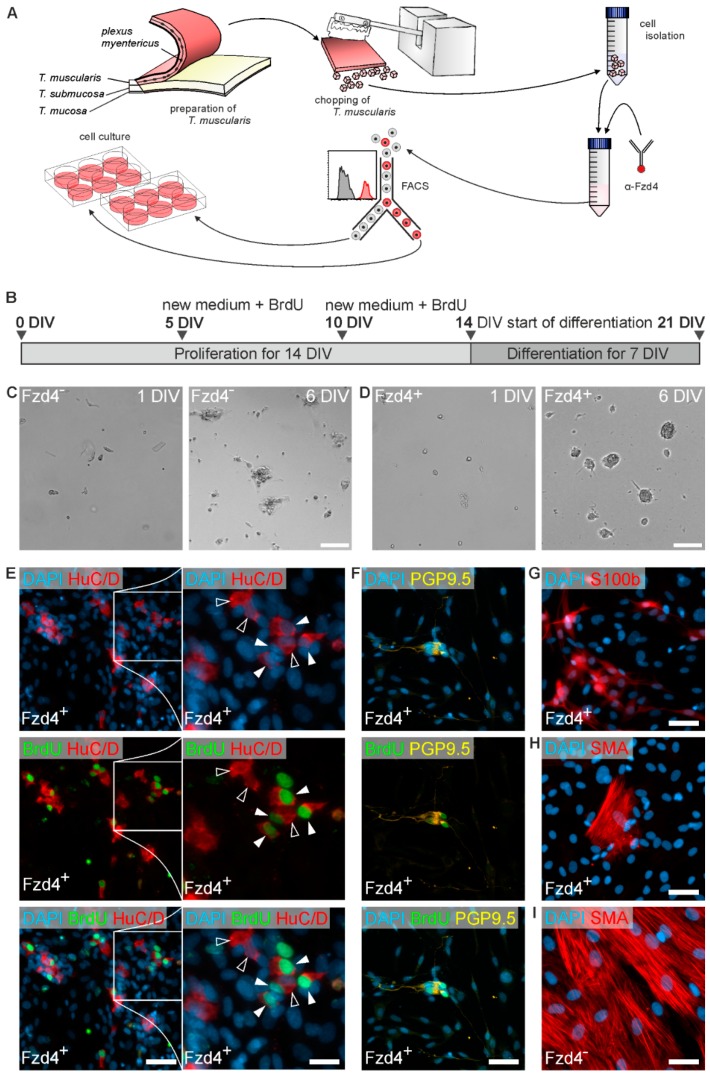
Neurosphere culture of Fzd4^+^ cells. (**A**) shows a scheme of the isolation and FACS procedures prior to culturing. *Tunica muscularis* of human resectates was detached from the other tissue layers and copped. Tissue parts were then digested with collagenase and dispase and isolated singe cells were stained with PE-conjugated anti-Fzd4 antibodies. FACS was used to separate Fzd4^+^ and Fzd4^−^ cell populations for subsequent culture. In (**B**) the timeline of the cell culture experiment is shown. Medium was replaced at 5 and 10 DIV (days in vitro) with BrdU being added on these days. After two weeks, we initiated cell differentiation. (**C**,**D**) show bright field micrographs of Fzd4^+^ and Fzd4^−^ cells during proliferation phase. Fzd4^+^ cells formed neurosphere-like bodies within 6 days *in vitro*, whereas most of the Fzd4^−^ cells largely grew adherent to the uncoated culture plastic. (**E–I**) depict immunostainings of the cell cultures after differentiation. Fzd4^+^ cells gave rise to neurons (HuC/D in (**E**), PGP9.5 in (**F**)), which incorporated BrdU during the proliferation phase (white arrowheads in the high power magnification in (**E**), hollow arrowheads show neurons without BrdU incorporation. Fzd4^+^ cultures also gave rise to S100b expressing glial cells (**G**). In Fzd4^−^ group no neurons or glia cells were detected. Instead, most cells were smooth muscle cells in Fzd4^−^ cultures ((**I**), SMA: smooth muscle actin), whereas there were only few smooth muscle cells in Fzd4^+^ cultures (**H**). Scale bars: 100 µm in bright field images; 50 µm in epifluorescence images and 20 µm in high magnification in (**E**).

After isolation and sorting (Figure 3A), both cell populations were cultured for two weeks under proliferation conditions, with BrdU being added together with every exchange of cell culture medium. Thereafter, we initiated differentiation and cultured the cells for an additional week (Figure 3B). Interestingly, frizzled-4^positive^ cells gave rise to free-floating spheroids during the proliferation phase (Figure 3D), whereas frizzled-4^negative^ cells exhibited a more adherent growth (Figure 3C). While we were not able to find any neural cells in the frizzled-4^negative^ cell pool after differentiation (Supplementary Figure S2), we identified neurons (HuC/D, PGP9.5; Figure 3E,F) and glial cells (S100b; Figure 3G) within the frizzled-4^positive^ culture by immunocytochemistry. Additionally, we found several cells co-labelled for both pan-neuronal markers and BrdU, indicating that these cells divided and successively differentiated into neurons in vitro (Figure 3E,F). It remains an open question when these neuronal differentiation markers are starting to be expressed during the in vitro culture. Frizzled-4^negative^ cultures, however, were largely dominated by smooth muscle cells (SMA, Figure 3I). Although we also found non-neural cells in frizzled-4^positive^ cultures (Figure 3H), these cells were few in number directly after seeding and continued to proliferate under differentiation cell culture conditions. It is noteworthy that frizzled-4 was not detected on smooth muscle cells in tissue sections (Figure 1A), strongly indicating that these few non-neuronal cells were erroneously sorted into frizzled-4^positive^ cultures due to minimal technical inaccuracy of the FACS process.

This result strongly supports the hypothesis that frizzled-4 is expressed by enteric neural progenitors, whereas frizzled-4^negative^ cells do not give rise to neurons or glial cells *in vitro*. Interestingly, this frizzled-4^negative^ population partly makes up the P75^NTR positive^ cell pool as reported by Nothelfer [25], indicating that not all P75^NTR^ expressing cells are capable of giving rise to neural cells. Our results therefore demonstrate that although frizzled-4 is not specific to neural stem/progenitor cells only, it still might by a more precise marker than the currently used P75^NTR^ as it does not exclude any neurogenic cells. It is noteworthy that more precise does not necessarily mean more effective or generally better, as frizzled-4 negative cells might still have a positive effect on the survival or the proliferative capacity of progenitor cells in vitro. Stable cultures of a small and precisely restricted cell population are in general harder to establish and more vulnerable in vitro compared to numerically larger, potentially less precisely sorted cell populations or even unpurified cultures.

Interestingly, we are not aware of any reports on the expression of frizzled-4 in neurogenic niches of the central nervous system. A recent work by Jin and colleagues, however, claims that frizzled-4 is crucial for stemness and invasiveness of glioma stem cells [29]. Additionally, frizzled-4 is a binding partner for the BMP-antagonist Norrin [30] and was reported reduce excitotoxic effects of NMDA on retinal ganglion cells via an activation of the canonical Wnt-pathway [31]. In this model, Müller cells also contributed to this neuroprotection by secretion of neurotrophic factors in response to a norrin-dependent canonical Wnt activation [31]. In addition, norrin is also known to regulate the establishment and maintenance of the blood-brain- and blood-retina-barrier and the vascularization of the inner ear via frizzled-4 receptors [32]. It is therefore conceivable that frizzled-4 in the ENS is not only expressed by neurogenic progenitors themselves, but also by enteric glia cells that serve as mediators for neuroprotection or pro-proliferative signalling.

### 3.4. Patch-Clamp Reveals Nav and K Channels in Frizzled-4^positive^ Sort

**Figure 4:**
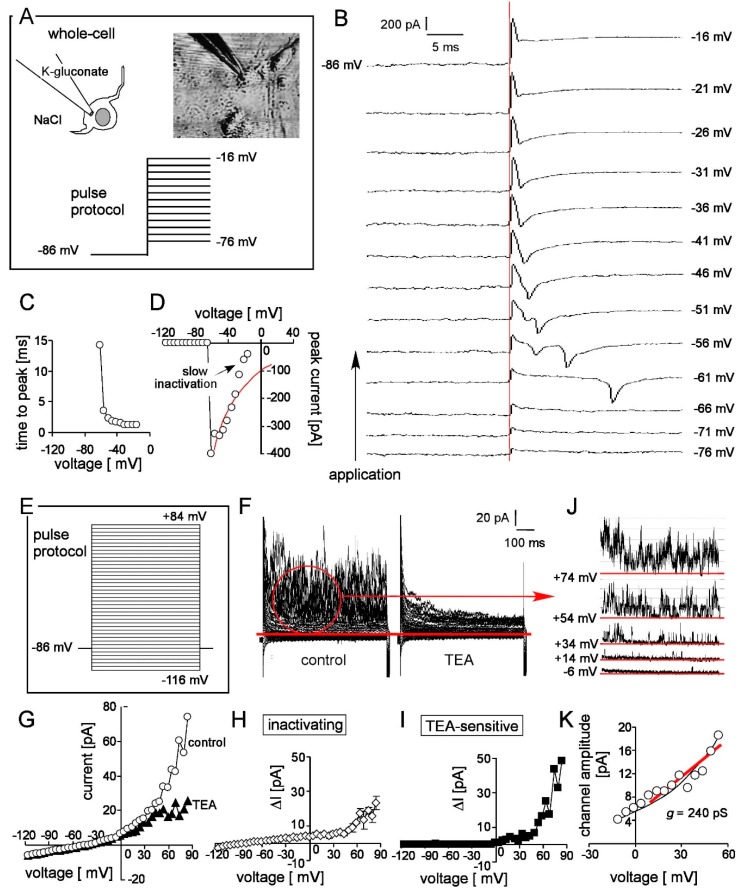
Patch clamp recording of Na_v_, K_v_ and BK channels in stem cell-derived enteric neurons. Experimental settings of whole cell recording (**A**), current tracings (**B**), voltage dependence of time-to-peak (**C**) and peak current amplitude (**D**) of Na_v_ channels. Applied pulse protocol (**E**), original current tracings (**F**) and current voltage relationship of K^+^-selective whole-cell currents recorded before ((**F**), left and (**G**), open circles) and during bath application of the K^+^ channel inhibitor tetraethylammonium (TEA, 3 mM, (**F**), right and (**G**), closed triangles). (**H**,**I**) Voltage dependence of the TEA-sensitive (**H**) and the time-dependent inactivating (**I**) current fractions. (**J**,**K**) Current tracings (**J**) and voltage dependence (**K**) of the unitary current transitions that underlie the TEA-sensitive K^+^ current. The red line in (**D**) indicates the theoretical current-voltage relationship of Na_v_ channels.

To test for functional differentiation, Na^+^- and K^+^-channels were characterized in expanded and differentiated frizzled-4^positive^ cells by whole-cell voltage-clamp recording (Figure 4A,E). Depolarizing voltage sweeps evoked time-dependent activating and fast inactivating inward Na^+^-currents (Figure 4B,C). Beyond fast inactivation, these currents inactivated slowly during the first train of depolarizing voltage sweeps (Figure 4B,D). Voltage-gating, fast and slow inactivation are hallmarks of voltage-gated Na_v_-channels [33]. In addition to Na_v_, TEA (3 mM)-insensitive, time-dependent inactivating (Figure 4F–H) and TEA-sensitive outwardly rectifying K^+^-selective currents (Figure 4F,G,I) were apparent. The former showed typical features of A-type K_v_-currents [34]. The channels underlying the TEA-sensitive K^+^-currents increased open probability with increasing positive voltage (Figure 4J) and exhibited unitary conductances of >200 pS (Figure 4K) reminiscent of large conductance Ca^2+^-activated BK K^+^-channels [35].

Combined, these data indicate activity of Na_v_, K_v_ and BK channels and, hence, suggest functional differentiation into neurons.

## 4. Conclusions

Our data provide strong evidence that the Wnt-receptor frizzled-4 is a useful marker for the isolation of postnatal neural progenitor cells from the human gut. It also supports our previous findings that canonical Wnt signalling has an enhancing effect on proliferation of these cells in vitro [24]. Moreover, frizzled-4 is expressed by a subpopulation of P75^NTR positive^ cells, without excluding any neurogenic cells and therefore is more precise in the isolation of enteric progenitor cells. Future experiments should be directed towards unravelling the Wnt-related microenvironment in vivo and evaluating potential cross-talks with other signalling pathways, e.g., serotonin [36], GDNF [37], and BMPs [38]. Moreover, characterizing the co-expression of frizzled-4 with other potential stem cell markers in the neurogenic cell population will help to identify and characterize the enteric neuronal stem cell niche in the human intestine.

## Supplementary Materials

The following are available online at https://www.mdpi.com/2073-4409/8/8/792/s1. Supplementary Table S1: Listed are all specimens used in this study. Supplementary Table S2: Antibodies used in this study. Supplementary Figure S1. Ancestry analyses of Fzd4^+^ and Fzd4^−^ cell populations. Supplementary Figure S2. Lack of neural cells in Fzd4^−^ cell cultures.
