# Supplementary material for: Wnt Receptor Frizzled-4 as a Marker for Isolation of Enteric Neural Progenitors in Human Children
Source: Cells. 2019 Jul 30;8(8):792. doi: 10.3390/cells8080792 (PMC6721585; doi:10.3390/cells8080792)
Supplement: Supplementary file 1 [file cells-08-00792-s001.pdf]

Supplementary table 1: Listed are all specimens used in this study. Samples are gut segments unaffected by the initial diagnosis and resected in the course of stoma relocations.

Age	Gender	Gut region	Diagnosis	Experiments
6 month	female	colon	imperforate anus	FACS, proliferation, differentiation
40 month	female	colon	yolk sac tumor	FACS, proliferation, differentiation
7 month	female	colon	imperforate anus	FACS, proliferation, differentiation
9 month	male	colon	meconium ileus	FACS, proliferation, differentiation, patch-clamping
9 month	female	colon	imperforate anus	FACS, proliferation, differentiation
12 month	female	colon	imperforate anus	FACS, proliferation, differentiation
8 month	female	colon	imperforate anus	FACS, proliferation, differentiation
9 month	male	colon	imperforate anus	FACS, proliferation, differentiation
1 month	male	colon	Hirschsprung's disease *	histology
1.5 month	male	colon	Hirschsprung's disease *	histology
9 month	female	colon	Hirschsprung's disease *	unsorted enterospheres
4 month	female	ileum	meconium plug syndrome	unsorted enterospheres
5 month	male	colon	imperforate anus	unsorted enterospheres

* Samples were collected from normoganglionic gut segments.

Supplementary table 2: Antibodies used in this study

Epitope	Host	Dilution	Resource
**Fzd4**	mouse	1:20	BioLegend, San Diego, CA, USA
**Fzd4 ***	mouse	undiluted hybridoma supernatant	Nothelfer et al.[25]
**HuC/D**	mouse	1:50	Life technologies, Carlsbad, CA, USA
**PGP9.5**	mouse	1:300	BIO RAD, Puchheim, Germany
**S100b**	rabbit	1:400	Abcam plc, Cambridge, UK
**SMA**	rabbit	1:100	Spring Bioscience, Pleasanton, CA, USA
**BrdU**	rat	1:100	MorphoSys AbD GmbH, Düsseldorf, Germany
**anti-rabbit Cy3**	goat	1:400	Jackson Immuno Research, Newmarket, UK
**anti-rat Alexa488**	goat	1:500	Invitrogen, Carlsbad, CA, USA
**anti-mouse Alexa488**	goat	1:500	Invitrogen, Carlsbad, CA, USA

∗Monoclonal mouse anti-human antibody CH3A4 against frizzled-4 was raised by immunization with the retinoblastoma cell line WERI-RB-1 and specificity for frizzled-4 was verified by the selective recognition of HEK-293 cells transfected with human frizzled-4. This molecule was clustered to CD344 at the HCDM workshop in Quebec, Canada

(http://www.hcdm.org/). Kindly provided by Hans Jörg Bühring. This antibody is mechanized in purified form by BioLegend.

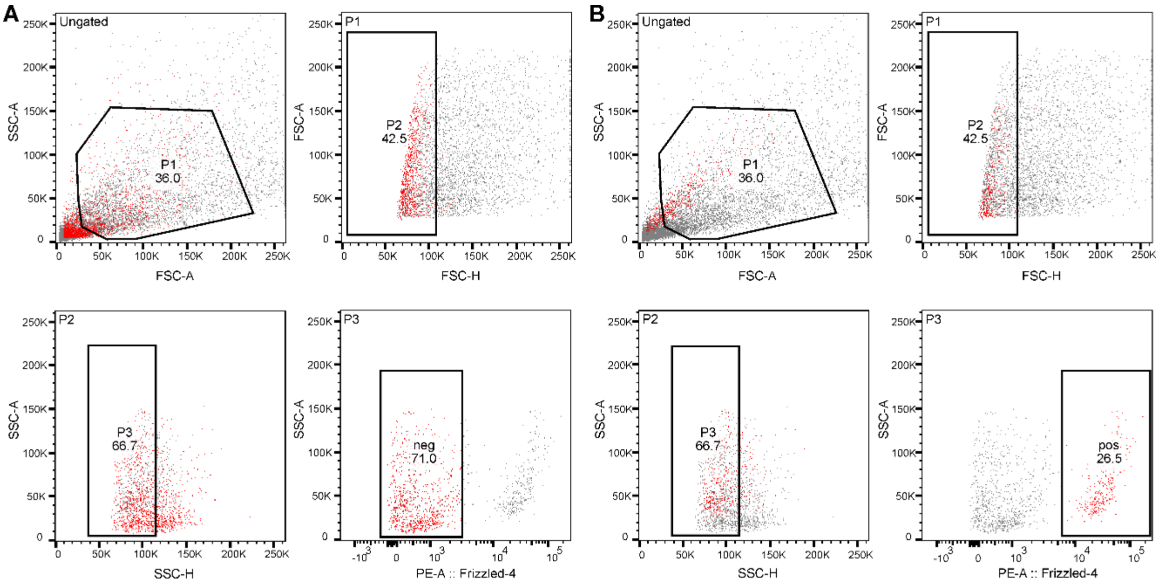

Supplementary Figure 1. Ancestry analyses of Fzd4$^+$ and Fzd4$^-$ cell populations. The scatter blot of the ancestry analyses of the representative experiment illustrated in Figure 2 are shown in red for Fzd4$^-$ (**A**) and Fzd4$^+$ (**B**) cells, respectively. This allows us to show whether the population of interest differs from the main population or exhibits a specific scatter profile. Thereby, we are able to identify the counts of Fzd4+ and Fzd4- cells in the scatter blots of the parent populations. There was no clear correlation of scattering pattern to Fzd4 expression detectable.

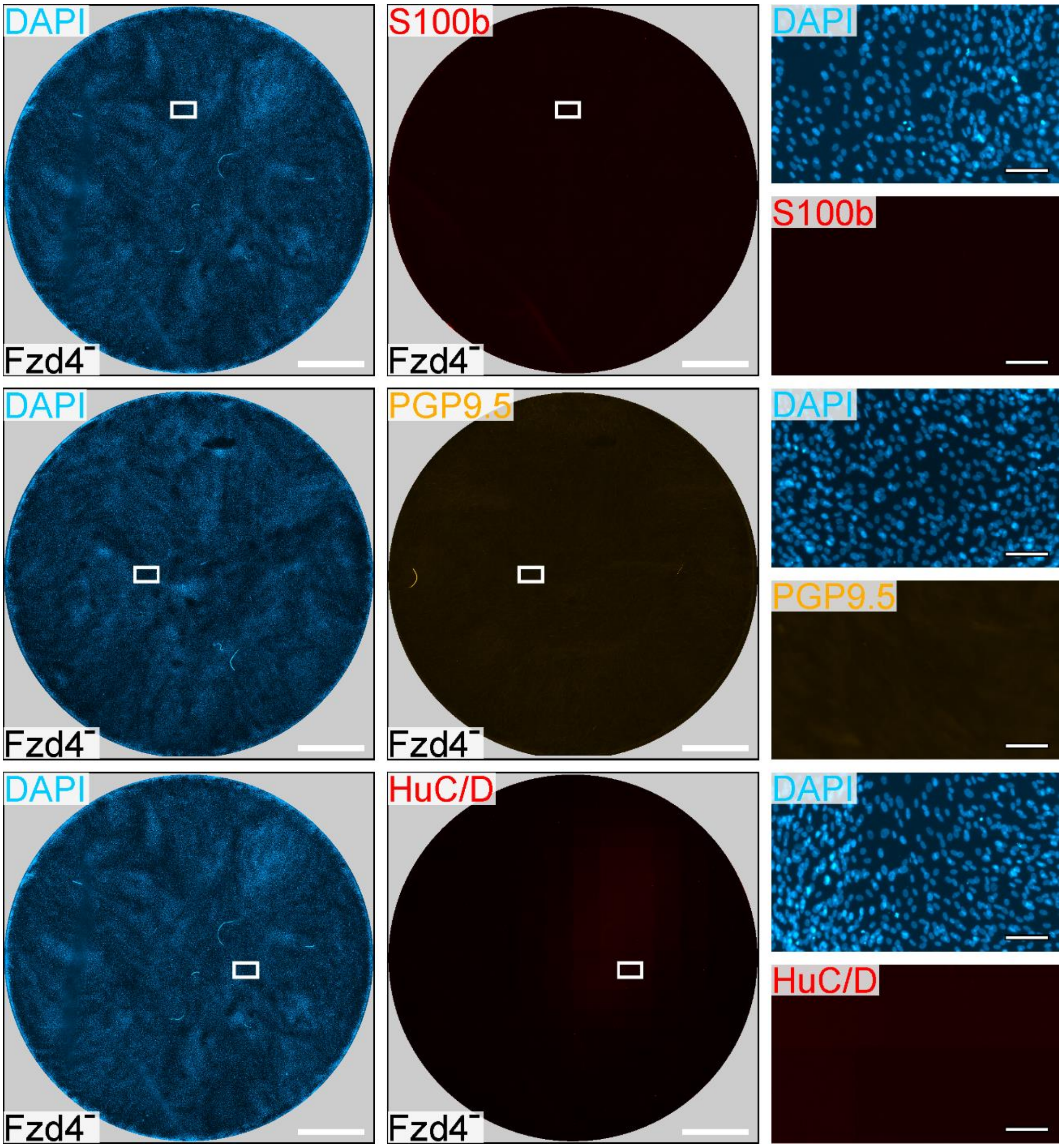

Supplementary Figure 2. Lack of neural cells in Fzd4$^-$ cell cultures. Shown are overviews over entire representative wells of Fzd4$^-$ cultures, as well as high resolution excerpts as indicated by the white rectangles. Stainings were performed for nuclei (DAPI) and for glial (S100b) or neuronal markers (PGP9.5, HuC/D). We did not detect a single neural cell in Fzd4$^-$ cultures. Scale bars: overviews 2 mm; detail excerpts 100 µm.
